# Changes in Vestibulo-Ocular Reflex Gain After Surgical Plugging of Superior Semicircular Canal Dehiscence

**DOI:** 10.3389/fneur.2020.00694

**Published:** 2020-07-21

**Authors:** Sang-Yeon Lee, Yun Jung Bae, Minju Kim, Jae-Jin Song, Byung Yoon Choi, Ja-Won Koo

**Affiliations:** ^1^Department of Otorhinolaryngology-Head and Neck Surgery, Seoul National University Bundang Hospital, Seongnam, South Korea; ^2^Department of Radiology, Seoul National University Bundang Hospital, Seongnam, South Korea; ^3^Seoul National University Bundang Hospital, Seoul National University College of Medicine, Seoul, South Korea

**Keywords:** superior semicircular canal dehiscence, plugging, vestibulo-ocular reflex, third mobile window, video head impulse test

## Abstract

Superior semicircular canal dehiscence (SCD), which is characterized by a “third mobile window” in the inner ear, causes various vestibular and auditory symptoms and signs. Surgical plugging of the superior semicircular canal (SC) can eliminate the symptoms associated with increased perilymph mobility due to the presence of the third window. However, the natural course of vestibular function after surgical plugging remains unknown. Therefore, we explored longitudinal vestibular function after surgery in 11 subjects with SCD who underwent SC plugging using the middle cranial fossa approach. Changes in vestibulo-ocular reflex (VOR) gain in all planes were measured over 1 year with the video head impulse test. We also evaluated surgical outcomes, including changes in symptoms, audiometric results, and electrophysiological tests, to assess whether plugging eliminated third mobile window effects. The mean VOR gain for the plugged SC decreased from 0.81 ± 0.05 before surgery to 0.65 ± 0.08 on examinations performed within 1 week after surgery but normalized thereafter. Four of seven subjects who were able to perform both VOR tests before surgery and immediately after surgery had pathologic values (SC VOR gain < 0.70). Conversely, the mean VOR gain in the other canals remained unchanged over 1 year. The majority of symptoms and signs were absent or markedly decreased at the last follow-up evaluation, and no complications associated with the surgery were reported. Surgical plugging significantly attenuated the air-bone gap, in particular at low frequencies, because of increased bone conduction thresholds and deceased air conduction thresholds. Moreover, surgical plugging significantly increased vestibular-evoked myogenic potential thresholds and decreased the ratio of summating potential to action potential in plugged ears. Postoperative heavily T2-weighted images were available for two subjects and showed complete obliteration of the T2-bright signal intensity in the patent SC lumen in preoperative imaging based on filling defect at the site of plugging. Our results suggest that successful plugging of dehiscent SCs is closely associated with a transient, rather than persistent, disturbance of labyrinthine activity exclusively involved in plugged SCs, which may have clinical implications for timely and individualized vestibular rehabilitation.

## Introduction

Superior semicircular canal dehiscence (SCD), which is characterized by a “third mobile window” in the inner ear, presents with debilitating vestibulo-cochlear symptoms due to bony dehiscence in the superior canal (SC) (1). The “third mobile window” in the otic capsule is frequently seen in the arcuate eminence facing the middle cranial fossa dura or occasionally seen in the SC close to the common crus by the superior petrosal sinus (2). This pathologic third mobile window increases the vestibular response to various stimuli, such as sound, pressure, and skull vibration (3, 4). Increased sensitivity to bone-conducted sounds can present as autophony and pulsating tinnitus. In addition to subjective symptoms and radiologic evaluation, objective demonstration of cochleo-vestibular hyperresponsiveness can be performed using vestibular-evoked myogenic potentials (VEMP), electrocochleography (ECoG), bone conduction audiometry, and pathologic vestibulo-ocular reflex (VOR) measurements induced by various stimuli. Surgical plugging of the dehiscent SC alleviates the aforementioned symptoms (5–8).

A meta-analysis confirmed that surgical plugging of the dehiscent SC significantly relieves subjective vestibular symptoms in patients with SCD (9). Although the exact mechanism underlying this effect remains unknown, the correction of hypermobile fluid dynamics in the otic capsule by blocking the pressure shunt into the vestibular system may be involved (10, 11). However, surgical plugging in the dehiscent SC may disturb natural fluid dynamics in all semicircular canals (SCCs). Recently, early quantitative video head impulse tests performed within 1 week after surgery showed a tendency for vestibular hypofunction in all ipsilateral canals and some contralateral canals as well as the emergence of compensatory saccades at an early phase (11). The severely reduced VOR gain for plugged SCs remained unchanged over time, whereas the VOR function in other canals generally, if not always, resolved after surgery (11). Nevertheless, the limitations of previous studies, such as relatively short follow-up periods and small sample sizes, render the associations between changes in vestibular function and surgical repair of the SCD speculative and presumptive, in particular for long-term vestibular function. Indeed, no data have been collected on the natural course of vestibular function after surgical plugging of dehiscent SCs.

Therefore, we explored longitudinal vestibular function after surgical plugging via the middle cranial fossa approach in subjects with SCD by measuring changes in VOR gains in all SCCs over time with video head impulse tests. We also evaluated the surgical outcomes of these subjects based on their subjective symptoms, audiometric results, and electrophysiological test findings to assess whether surgical plugging eliminated pathologic third mobile window effects. We observed an immediate deterioration of VOR gain in plugged SCs that stabilized thereafter in the majority of cases, although preserved VOR gains persisted in the other canals. Our results suggest a differential progressive nature of VOR gain between plugged and non-plugged canals following surgical plugging of dehiscent SCs, which paves the way for understanding changes in vestibular function induced by canal plugging in subjects diagnosed with SCD.

## Methods

### Participants

The medical records of subjects diagnosed with SCD at Seoul National University Bundang Hospital between January 2015 and December 2019 were retrospectively reviewed. The diagnostic criteria of SCD were based on the combination of dehiscent SC based on high-resolution temporal bone computed tomography (HR-TBCT) images reformatted in the plane of the SC, symptoms and signs relevant to third window syndrome, and at least one objective source documenting abnormal pressure transmission via a third mobile window (1). This study included only subjects in whom SC plugging was performed by a single surgeon (J.W.K.) via a middle fossa approach. Subjects who underwent a video head impulse test before surgery and at least one video head impulse test after surgery were selected to obtain comparison data. Ultimately, 11 subjects were identified. None of the subjects had a history of brain surgery or Meniere's disease, head injury, or neurological disorders. This study was approved by the Seoul National University Bundang Hospital Institutional Review Board (IRB No. B-2004-604-125) and was conducted in accordance with the principles of the Declaration of Helsinki.

### Surgical Intervention

Canal plugging was performed with the aim of blocking the abnormal pressure shunt toward the third mobile window. The dehiscent SC was occluded with a combination of soft tissue and bone wax to obtain a watertight seal and then covered with temporalis fascia using the middle cranial fossa approach (Video S1).

### Video Head Impulse Test: Follow-Up Protocol and Parameters

Head impulse tests were performed with a video system for the acquisition and analysis of eyeball and head movements (ICS Impulse®, GN Otometrics, Denmark), as described in our previous studies. For collection of head impulse data, 20 is the recommended minimum number of head impulses in the standard protocol outlined by the manufacturer. In some cases where we encountered difficulties in collecting acceptable stimulations, video head impulse test responses were analyzed from at least 10 acceptable impulse stimulations. As such, the tests were repeated at least 10 times on each side in an unpredictable direction, using the center-to-outward rotation method, at 5–10° and with peak accelerations of 750–6000°/s (12). Only artifact-free recordings with sufficient head velocity were used for further analyses. The movements of the right eyeball and head were recorded. VOR gain in the video head impulse tests was defined as the ratio of the area under the velocity curves of the right eye to that of the head (13). According to previous studies (14, 15), normal VOR gain is specified by the video head impulse test (ICS Impulse®, GN Otometrics, Denmark) as >0.8 for the lateral canals and >0.7 for the vertical canals. Subjects who underwent surgical plugging were instructed to undergo serial video head impulse tests before surgery; immediately after surgery (within 1 week); and at 2, 6, and 12 months after surgery, if available.

### Pure-Tone Audiometry

All subjects underwent pure-tone audiometry testing before and after surgery. In a soundproof booth, the pure-tone thresholds of bone and air conduction were recorded with standard audiometric testing procedures (ANSI, 1978, New York). The mean hearing threshold was calculated with the average of the hearing thresholds at 0.5, 1, 2, and 4 kHz. Masking was used with the air conduction threshold test if necessary and was used routinely with the bone conduction threshold test in the contralateral ear. Preoperative audiograms were performed an average of 1 day before surgery. The last available postoperative audiogram was used for analyses. Four audiogram parameters were analyzed to obtain comparable data before and after surgery: bone conduction thresholds at 0.25, 0.5, and 1 kHz; air conduction thresholds at 0.25, 0.5, and 1 kHz; air-bone gaps (ABGs) across 0.25, 0.5, and 1 kHz; and ABGs across 0.5, 1, 2, and 4 kHz.

### Vestibular Evoked Myogenic Potentials

Cervical VEMPs (cVEMPs) were measured during ipsilateral sternocleidomastoid muscle (SCM) contraction. Subjects were placed in the supine position with their head raised ~30° from the horizontal and rotated contralaterally to maintain contraction of the ipsilateral SCM. We recorded surface electromyography (EMG) activity from an active electrode placed over the belly of the contracted SCM after subtracting EMG activity from a reference electrode located on the medial clavicle. A ground electrode was attached to the forehead. Alternating tone bursts (500 Hz; rate, 2.1/s; rise-fall time, 2 ms; plateau time, 3 ms; 128 repetitions; Navigation Pro; Biologic Systems, Mundelein, IL, USA) were provided to each ear. The analysis time for each stimulus was 50 ms, and responses elicited by up to 80 stimuli were averaged for each test. We determined the thresholds by lowering the sound stimulus from the 93 dB normalized hearing level (nHL) in 5 dB decrements.

### Electrocochleography

Extratympanic ECoG was recorded with a commercial acoustic evoked potential unit (Navigation Pro ver. 7.0.0; Biologic Systems). ER3-26B gold Tiptrodes (Etymotic Research, Elk Grove, IL, USA) were placed close to the tympanic membrane in the external auditory canal. Stimuli consisting of alternating polarity clicks (band-pass filtered, 10e1500 Hz) of 100 ms duration were presented at an intensity of 90 dB nHL. Two replications of averaged responses elicited by 1000–1500 clicks at 7.1 per second were obtained. As mentioned previously, the amplitudes were measured from baseline to summating potential (SP) or action potential (AP) peaks to elicit the SP/AP ratios (16).

### Imaging Protocol

All subjects underwent a preoperative HR-TBCT to confirm the dehiscence of the SC. HR-TBCT was performed with a 64-section multidetector CT scanner (Brilliance; Phillips Healthcare, Best, The Netherlands) with the following parameters: collimation, 40 × 0.625 mm; slice thickness, 0.67 mm; increment, 0.33 mm; pitch, 0.825, 120 kVp, 250 mAs. As described by previous reports, images were displayed on an INFINITT PACS system version 3.0.9.1BN9 (INFINITT Healthcare, Seoul, Korea), and 3D multiplanar reconstruction was subsequently used to obtain an oblique coronal reformation image parallel to the SC. The length of the arc of dehiscence, which accounts for SCD size, was measured on a reformatted image in the plane of the SC.

Magnetic resonance imaging (MRI) examinations were performed on a 3 T MR scanner (Ingenia; Philips Healthcare) with a 32-channel SENSE Head Coil (Philips Healthcare). Heavily T2-weighted images (T2-WI) using 3D T2-weighted volume isotropic turbo spin-echo acquisition (T2-VISTA) were used to evaluate the SC structure. The imaging parameters were as follows: repetition time, 2000 ms; echo time, 258 ms; field of view, 160 × 160; acquisition matrix, 228 × 228; flip angle, 90°; echo train length, 74; number of excitations, 1; slice thickness, 0.70 mm; overlap, 0.35 mm. A three-dimensional multiplanar and/or maximum intensity projection (MIP) reconstruction with a slab 5 mm thick was used to evaluate the patency of the SC. MRI was performed at the 1-year follow-up after canal plugging in two subjects.

### Statistical Analyses

The data are presented as means ± standard errors of the mean (SEMs). All statistical analyses were performed and illustrated with R (R version 3.5.2 and R Studio 1.0.136, Foundation for Statistical Computing, Vienna, Austria). If the data were normally distributed, one-way within-subjects analysis of variance (ANOVA) and Tukey's *post hoc*-test were used to examine differences in VOR gain over time. In addition, independent *t*-tests were performed to compare cVEMP thresholds and SP/AP ratios between operated and normal ears. Furthermore, paired *t*-tests were used as appropriate to compare cVEMP thresholds, SP/AP ratios, and pure-tone audiometry before and after canal plugging. All statistical tests were two-tailed, and *P* < 0.05 was considered to indicate statistical significance.

## Results

### Demographic and Clinical Characteristics

The clinical characteristics of the 11 subjects enrolled in this study are summarized in Table 1. All 11 subjects underwent surgical plugging for SCD via the middle cranial fossa approach. Their mean age was 49.2 ± 2.97 years (range, 26–61 years), and seven were women. Temporal bone CT images reformatted in the plane of the SC revealed definite evidence of SCD in the affected ears, with a mean dehiscence size of the operated ears of 4.24 ± 0.23 mm (range, 3.07–5.48 mm). Eight subjects presented with unilateral SCD; the remaining three subjects presented with bilateral SCD. Subjects with bilateral SCD underwent canal plugging of the dominant SCD side only via the middle fossa approach. The SCD was located at the arcuate eminence in nine patients (10 sides) and at the level of the superior petrosal sinus in two patients.

**Table 1:** Demographics and clinical characteristics of 11 subjects with superior semicircular canal dehiscence.

**Case no**.	**Sex/age**	**Side**	**Dehiscence size (mm)^**a**^**	**Follow-up period^**b**^ (months)**	**Operation**	**Autophony**		**Ear fullness**		**Hearing loss**		**Dizziness**		**Tulio/Hennebert**		**Pulsating tinnitus**	
						**Pre**	**Post**	**Pre**	**Post**	**Pre**	**Post**	**Pre**	**Post**	**Pre**	**Post**	**Pre**	**Post**
1	F/58	R	3.18	46	R) plugging (MFA) T1^c^	NP	NP	NP	NP	•*	◦	•	◦	•	NP	•*	NP
2	F/61	R	3.93	44	R) plugging (MFA)	•*	NP	•	NP	NP	NP	NP	NP	NP	NP	NP	NP
3	M/56	L	4.01	51	L) plugging (MFA)	•*	NP	•	NP	•	◦	NP	NP	NP	NP	•	NP
4	M/26	B	4.33	37	L) plugging (MFA)	•	NP	•	NP°	NP	NP	•*	◦	•	NP	•	NP
5	M/47	B	3.9	30	L) plugging (MFA)	•*	NP	•	NP	NP	NP	•	NP	•	NP	•	NP
6	F/45	R	3.07	25	R) plugging (MFA)	•	NP	NP	NP	NP	NP	•*	◦	•	NP	•	◦
7	F/40	B	4.35	11	R) plugging (MFA)	•*	NP	•	NP	NP	NP	•	NP	NP	NP	•	◦
8	F/49	L	5.48	9	L) plugging (MFA) SPS encasing	•*	NP	•	NP	NP	NP	NP	NP	NP	NP	•	NP
9	M/53	L	4.98	7	L) plugging (MFA)	•*	NP	•	NP	NP	NP	•	◦	NP	NP	•*	NP
10	F/50	L	4.13	5	L) plugging (MFA) SPS encasing	•*	◦	•	NP	•	◦	•	NP	NP	NP	•	◦
11	M/56	L	5.24	3	L) plugging (MFA)	•*	NP	NP	NP	NP	NP	•	NP	NP	NP	•	NP

M, male; F, female; R, right; L, left; B, bilateral; MFA, middle fossa approach; SPS, superior petrosal sinus; NP, not present; •, present/ no change; °, improved; ^*^, chief complaint.

a *Note that the length of the arc of superior semicircular canal dehiscence, which accounts for dehiscence size, was measured on a reformatted image in the plane of the superior semicircular canal*.

b *Note that refers to period of follow-up from the surgery to the present. The status if postoperative symptoms after surgery is based on the present*.

c *Note that the case No.1 accompanies chronic otitis media with tympanic membrane perforation. Surgical plugging via middle cranial fossa approach was performed following endaural tympanoplasty type I*.

### Subjective Symptoms Before and After SC Plugging

As summarized in Table 1, of the preoperative cochleovestibular symptoms, autophony and pulsatile tinnitus were the most common (90.9% of subjects), followed by ear fullness (72.7%), dizziness (72.7%), Tulio/Hennebert signs (36.4%), and subjective hearing loss (27.3%). Only one subject (subject 1) did not report autophony before surgery, and it is interesting that she had a small tympanic membrane perforation (Figure S1).

The average number of debilitating symptoms per subject was three (range, 2–5). After surgery, the median follow-up time was 25 months (range, 3–51 months). No subject had postoperative complications. Most symptoms were absent or markedly relieved at the last follow-up evaluation. In the three subjects with bilateral SCD, lateralized symptoms resolved after surgery, but dizziness persisted in two patients, even after the dominant SC was plugged. During the follow-up period, we suggested that subjects who complain of dizziness after surgery should not be restricted through individual counseling. Specifically, the exercise-based program primarily designed to reduce vertigo, dizziness, and gaze instability, which consists of habituation, gaze stabilization, and balance testing, has not been prescribed to all subjects. Of them, two subjects enrolled in this study (Subject 1 and 3) experienced posterior canal benign paroxysmal positional vertigo (BPPV) in the affected ear for more than 1 year after surgery, which did not seem to be relevant to plugging surgery. All subjects returned to their normal activity.

### Longitudinal Changes in VOR Function After SC Plugging

**Figure 1:**
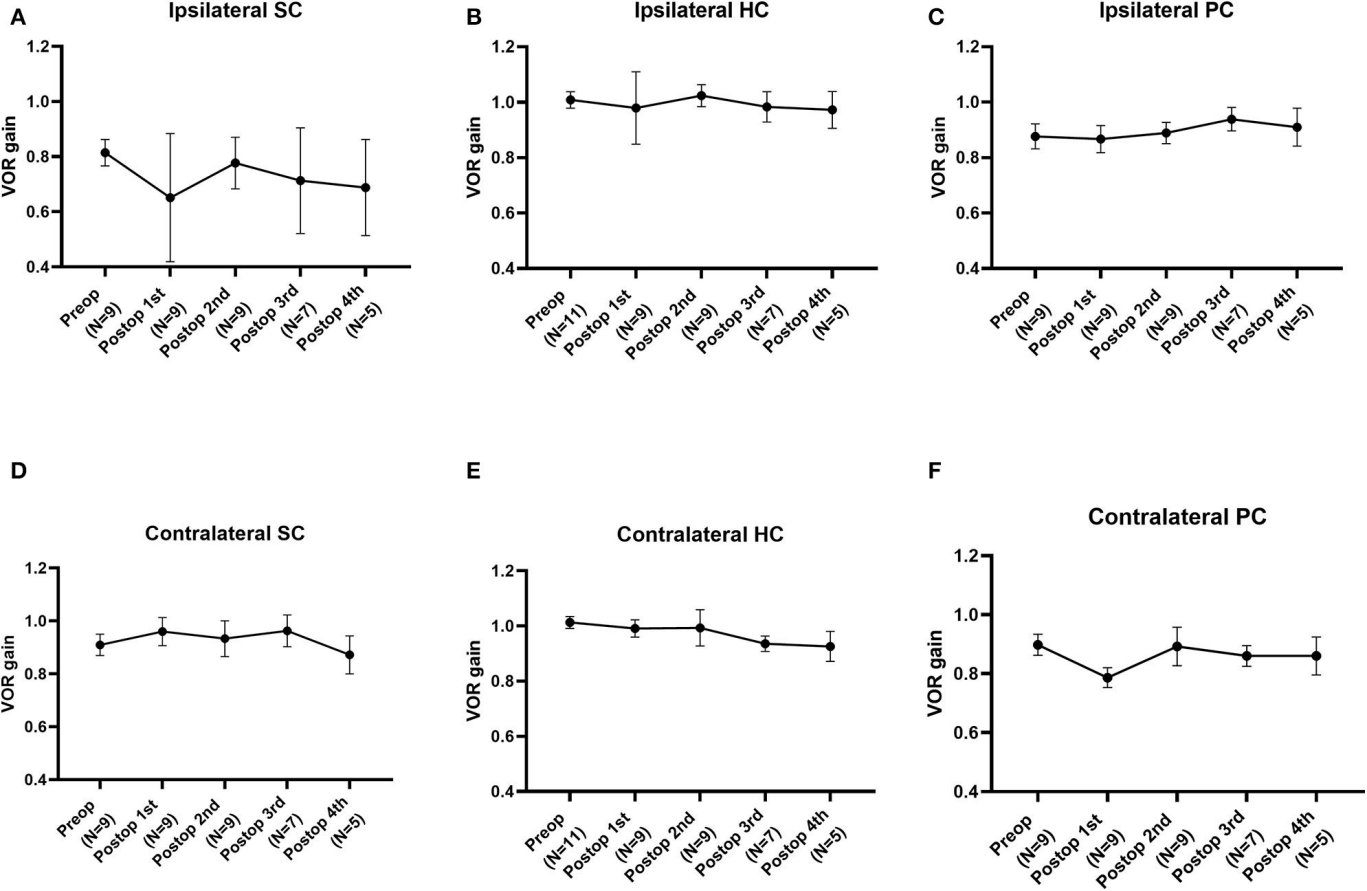
The natural course of the mean vestibulo-ocular reflex (VOR) gain for each semicircular canal (SC) over 1 year before and after plugging of the superior SC. **(A)** The mean VOR gain for plugged SCs decreased immediately within 1 week after surgery but improved over time, similar to the preoperative value. **(B–E)** Compared to the preoperative value, the mean VOR gain for each SC after canal plugging remained unchanged over 1 year of follow-up, without any significant changes between intervals. Data are means ± standard error of the means (SEMs).

**Figure 2:**
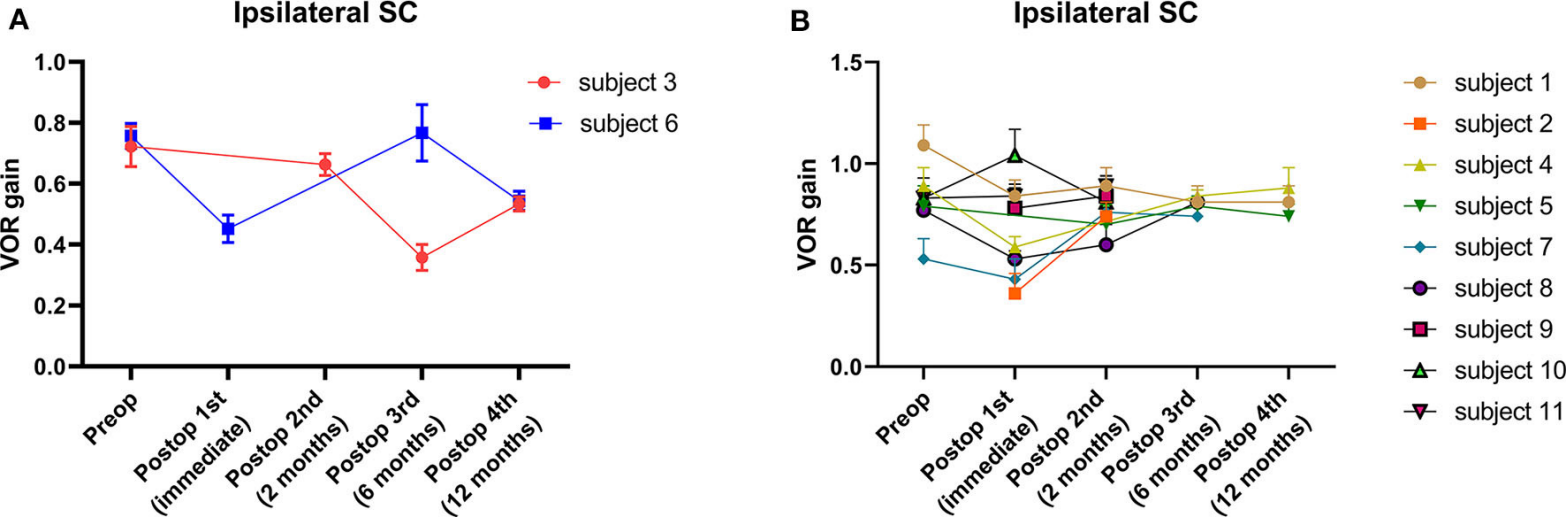
Individual changes in VOR gain for plugged SCs. **(A)** A fluctuating pattern of VOR gain for plugged SCs over time was observed in two subjects. The subjects showed a markedly decreased VOR gain at the 1-year (fourth postoperative) evaluation. **(B)** The typical pattern of VOR gain for plugged SCs over time in nine subjects. Compared to baseline, VOR gains were lower immediately after surgery (first postoperative evaluation) in most cases. Nonetheless, VOR gains for plugged SCs normalized over 1 year, similar to baseline values. In addition, no significant differences in mean VOR gains were observed between intervals at the second, third, and fourth postoperative evaluations. Data are means ± SEMs.

The average VOR gains over 1 year of six canals before and after SC plugging are shown in Figure 1. Of the 11 subjects, two had preoperative video head impulse test data from the lateral canals. Before surgery, no significant difference in gain was observed between the operated and normal ears for the SC, HC, and PC. The mean VOR gain of the plugged SCs decreased from 0.81 ± 0.05 before surgery (preoperative evaluation) to 0.65 ± 0.08 on examinations performed within 1 week after surgery (first postoperative evaluation). This represents an approximate 20% attenuation, although this difference was not statistically significant (95% confidence interval [CI] = −0.36 to 0.03, *P* = 0.092). Four of the seven subjects who underwent both the preoperative and first postoperative VOR tests contributed to the abnormal gain seen. The mean duration between the preoperative and first postoperative evaluations was 4.2 ± 0.4 days (range, 3–6 days). Compared to the first postoperative evaluation, the mean SC gain increased to 0.78 ± 0.03 during the second postoperative evaluation, a value similar to the preoperative value. The average time to the second postoperative evaluation was 63 ± 3 days (range, 48–78 days). VOR gain for the plugged SC remained unchanged thereafter (third postoperative evaluation, 0.71 ± 0.07; fourth postoperative evaluation, 0.69 ± 0.08). The average time to the third and fourth postoperative evaluations was 184 ± 6 days (range, 169–215 days) and 376 ± 6 days (range, 355–391 days), respectively. Likewise, individual data showed that the immediate plugged SC gain deteriorated by 1 week after surgery but subsequently normalized thereafter in most subjects, apart from two subjects (subjects 3 and 6; Figure 2). VOR gains for plugged SCs in these two subjects exhibited a fluctuating pattern over time. The subjects showed a markedly decreased VOR gain at the 1-year mark (fourth postoperative evaluation).

Except for the plugged SCs, the mean VOR gain for the semicircular canals did not differ significantly between intervals for each canal, demonstrating nearly equivalent values over 1 year. Although VOR gain in the contralateral PC tended to decrease from the preoperative to the immediate postoperative time point, likely recapitulating VOR gain in the plugged SC, the difference was not statistically significant. In addition, as documented by individual data, no subject had abnormal VOR gain in the contralateral PC that deviated from the normal value throughout the follow-up evaluations. Specifically, one subject (subject 8) exhibited an immediate postoperative reduction in VOR gain in the plugged SC as well as the ipsilateral HC. Although the ipsilateral HC gain was fully recovered thereafter, the reduced VOR gain in the plugged SC remained unchanged at the second postoperative evaluation.

### Audiological Characteristics Before and After SC Plugging

**Figure 3:**
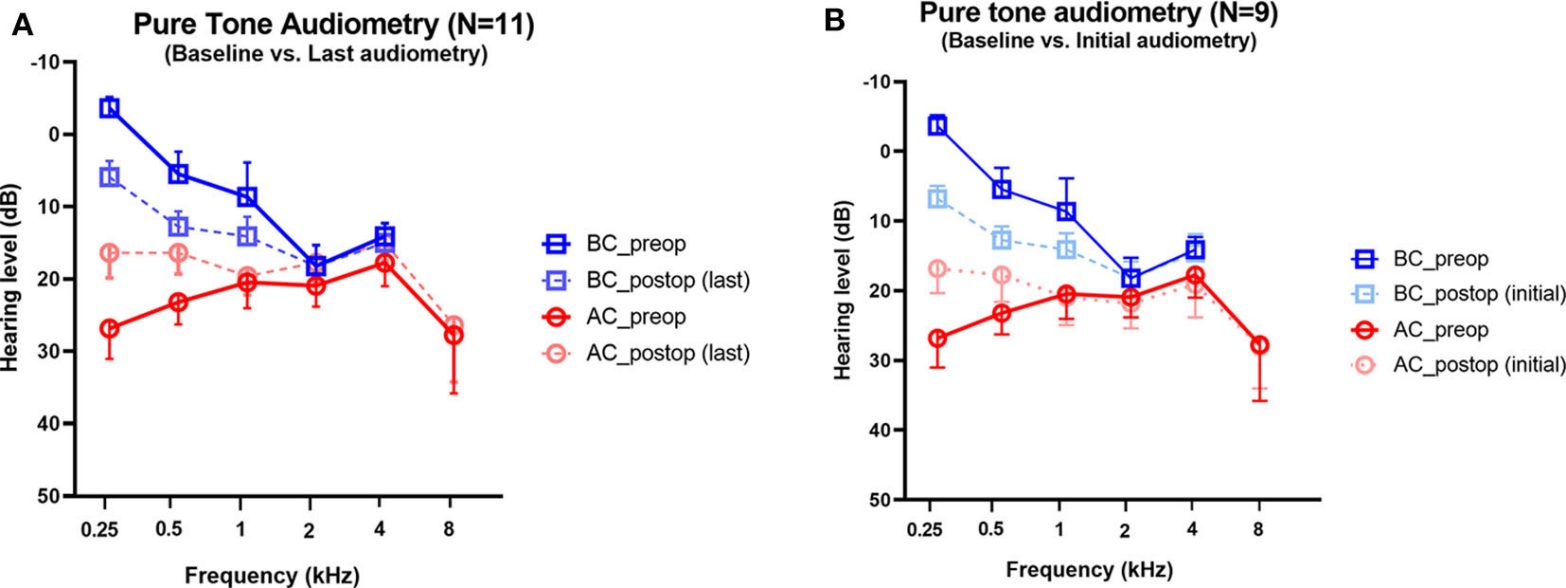
Changes in pure-tone audiometry in the operated ears. Preoperative and postoperative bone conduction and air conduction thresholds are shown for all tested frequencies. **(A)** The postoperative values are based on the last available audiogram. The average interval between baseline and the last audiogram was 226 days (range, 33–391 days). **(B)** The postoperative values are based on the initial available audiogram obtained between 1 and 3 months after plugging surgery. The average interval between baseline and the first audiogram obtained 3 weeks after surgery was 60 days (range, 27–84 days). For two subjects (Subject 7 and 8), the first audiometry assessments were conducted at 5 and 6 months, respectively; thereby, pure tone audiometry data obtained between 1 and 3 months after surgery were available for only nine subjects. Data are means ± SEMs. After surgery, air-bone gaps at 250 and 500 Hz improved significantly (*P* < 0.001, paired *t*-test).

Audiometry was performed before surgery and after surgery in all subjects. The average interval between baseline and the last audiogram was 226 days (range, 33–391 days). The mean bone and air conduction thresholds before and after surgical plugging are shown in Figure 3. Preoperative audiograms showed significant ABGs in the operated ears, in particular at low frequencies. Note that hypersensitive bone conduction thresholds (<0 dB HL) were observed in six subjects (54.5%) at 250 Hz, two subjects (18.2%) at 500 Hz, and one subject (5.6%) at 1 kHz. Compared to baseline, the average ABGs across 0.25, 0.5, and 1 kHz decreased significantly from 20.0 ± 2.6 to 6.5 ± 2.4 after surgery (95% CI = −20.8 to −6.2, *P* = 0.002), whereas the average ABGs across 0.5, 1, 2, and 4 kHz decreased significantly from 9.0 ± 2.1 to 2.2 ± 0.8 after surgery (95% CI = −12.39 to −1.24, *P* = 0.021) (Figure 3A). The bone conduction thresholds at 0.25, 0.5, and 1 kHz increased following surgery, from −3.6 ± 1.5 to 5.9 ± 2.2 (*P* < 0.001), 5.5 ± 3.1 to 12.7 ± 2.1 (*P* = 0.009), and 8.6 ± 4.7 to 14.1 ± 2.7 (*P* = 0.119), respectively. Moreover, only one subject showed bone conduction below 0 dB HL at 0.25 kHz after surgery. In addition, the air conduction thresholds at 0.25, 0.5, and 1 kHz following surgery decreased from 26.8 ± 4.2 to 16.4 ± 3.5 (*P* = 0.029), 23.2 ± 3.1 to 16.4 ± 3.0 (*P* = 0.027), and 20.5 ± 3.6 to 19.6 ± 2.7 (*P* = 0.690), respectively. At the higher frequency range, 15 dB hearing loss at 8 kHz was observed in subject 2 only. Otherwise, significant hearing loss at high frequencies was not observed during follow-up. Collectively, surgical plugging significantly attenuated ABGs, in particular at low frequencies, via increased bone conduction thresholds and decreased air conduction thresholds.

To support our findings with regard to the natural course of audiological recovery, we evaluated the audiological changes with reference to the initial audiogram obtained between 1 and 3 months after plugging surgery. The average interval between baseline and the first audiogram obtained 3 weeks after surgery was 60 days (range, 27–84 days). Unfortunately, pure tone audiometry data obtained between 1 and 3 months after surgery were available for only nine subjects. For the remaining two subjects (Subject 7 and 8), the first audiometry assessments were conducted at 5 and 6 months, respectively. In comparison with the baseline, the average ABGs across 0.25, 0.5, and 1 kHz decreased significantly from 20.0 ± 2.6 to 6.5 ± 2.4 at an average of 60 days (range, 27–84 days) after surgery (95% CI = −15.3 to −10.2, *P* < 0.001) (Figure 3B). When comparing audiological results between the initial and the last audiometry assessments, the average ABGs across 0.25, 0.5, and 1 kHz decreased significantly from 7.3 ± 3.1 to 6.5 ± 2.4 after surgery (95% CI = −3.3 to 1.7, *P* = 0.506). In addition, audiological profiles, which included average ABGs across 0.5, 1, 2, and 4 kHz, bone conduction thresholds, and air conduction thresholds at each frequency, did not differ between the initial and last audiometry assessments. Thus, surgical plugging significantly attenuated the air-bone gap, especially at low frequencies, even from the early phase after surgery, and the effects remained unchanged over time.

### cVEMP Thresholds and SP/AP Ratios After SC Plugging

**Figure 4:**
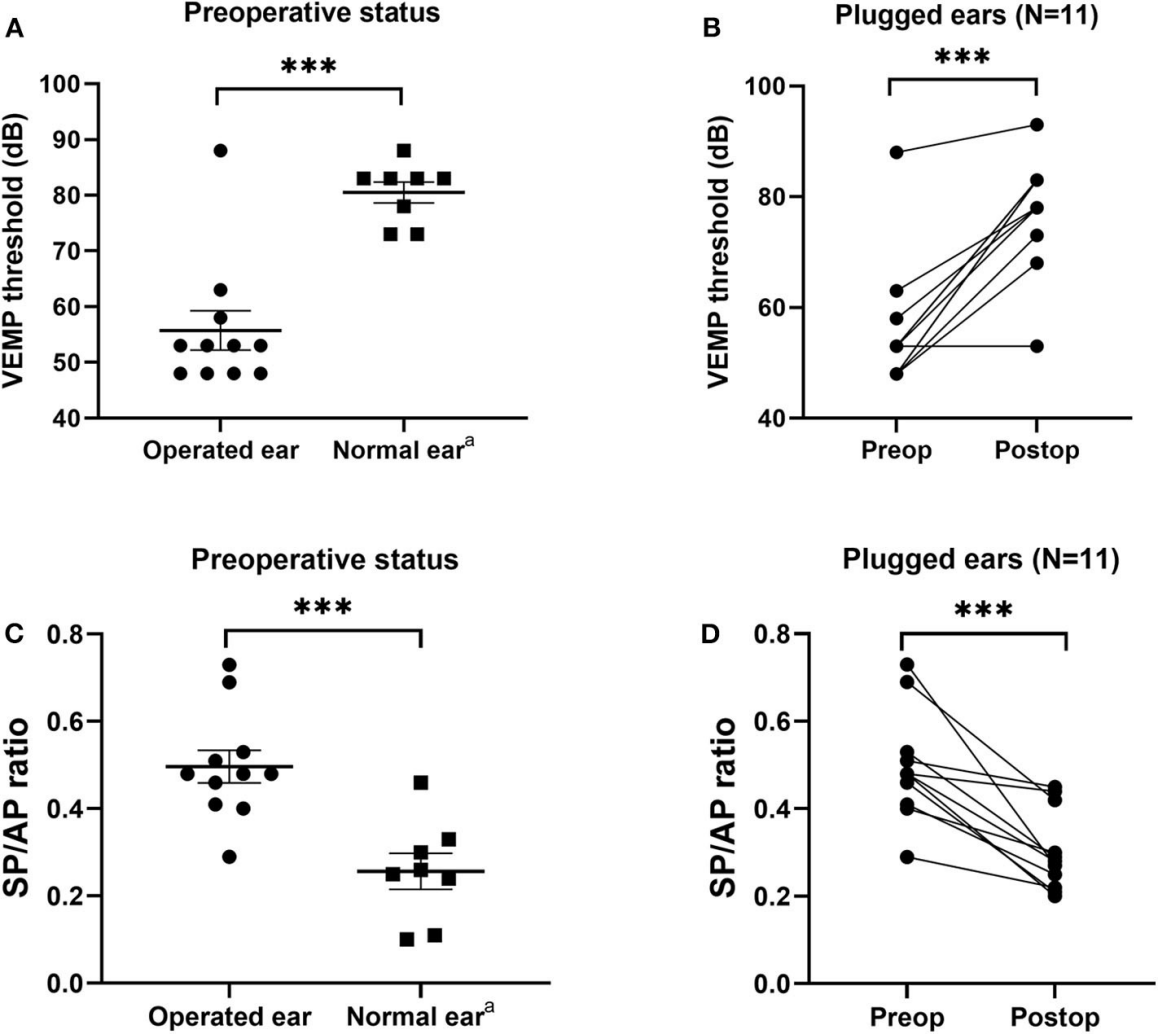
Electrophysiological findings before and after canal plugging. **(A)** Before surgery, the cervical vestibular evoked myogenic potential (cVEMP) threshold differed significantly between operated and normal ears (*P* < 0.001, independent *t*-test). **(B)** Mean cVEMP thresholds before and after surgery for all subjects. Preoperative and postoperative gain values for the same subject are connected by a line. At 2–3 months after surgery, the mean cVEMP thresholds in operated ears increased significantly (*P* < 0.001, paired *t*-test). **(C)** Before surgery, the ratio of summating potential to action potential (SP/AP) differed significantly between operated and normal ears (*P* < 0.001, independent *t*-test). **(D)** SP/AP ratios before and after surgery for all subjects. Preoperative and postoperative gain values for the same subject are connected by a line. At 2–3 months after surgery, the mean SP/AP ratio for operated ears increased significantly (*P* < 0.001, paired *t*-test). Data are means ± SEMs. ***P < 0.001. ^a^Note that three subjects with bilateral semicircular canal dehiscence (SCD) were excluded from the normal ear analyses.

All subjects underwent pre- and postoperative cVEMPs and ECoG. The interval between pre- and postoperative evaluations was ~2 months. As shown in Figure 4, mean cVEMP thresholds in the operated and normal ears were 55.7 ± 3.5 dB and 78.0 ± 2.2 dB (95% CI = 13.3 to 31.2, *P* < 0.001), respectively, before surgery and 77.6 ± 3.1 dB and 81.5 ± 2.1 dB (95% CI = −4.1 to 12.0, *P* = 0.317), respectively, after surgery. That is, SC plugging significantly enhanced the mean cVEMP thresholds in the operated ears (95% CI = 14.1 to 29.5, *P* < 0.001). Consistent with this, the mean SP/AP ratio in the operated ears decreased significantly from 0.50 ± 0.04 before surgery to 0.30 ± 0.02 after surgery (95% CI = −0.28 to −0.11, *P* < 0.001). Moreover, mean SP/AP ratios did not differ between operated and normal ears after surgery (95% CI = −0.13 to 0.05, *P* = 0.381).

One subject with tympanic membrane perforation (subject 1) exhibited normal cVEMP threshold and SP/AP ratio before surgery. After successful plugging and tympanoplasty, the cVEMP thresholds and SP/AP ratio remained within normal ranges, whereas the ABG disappeared and the bone conduction threshold normalized (Figure S1).

### MRI Findings: Structural Changes in SCs After Canal Plugging

**Figure 5:**
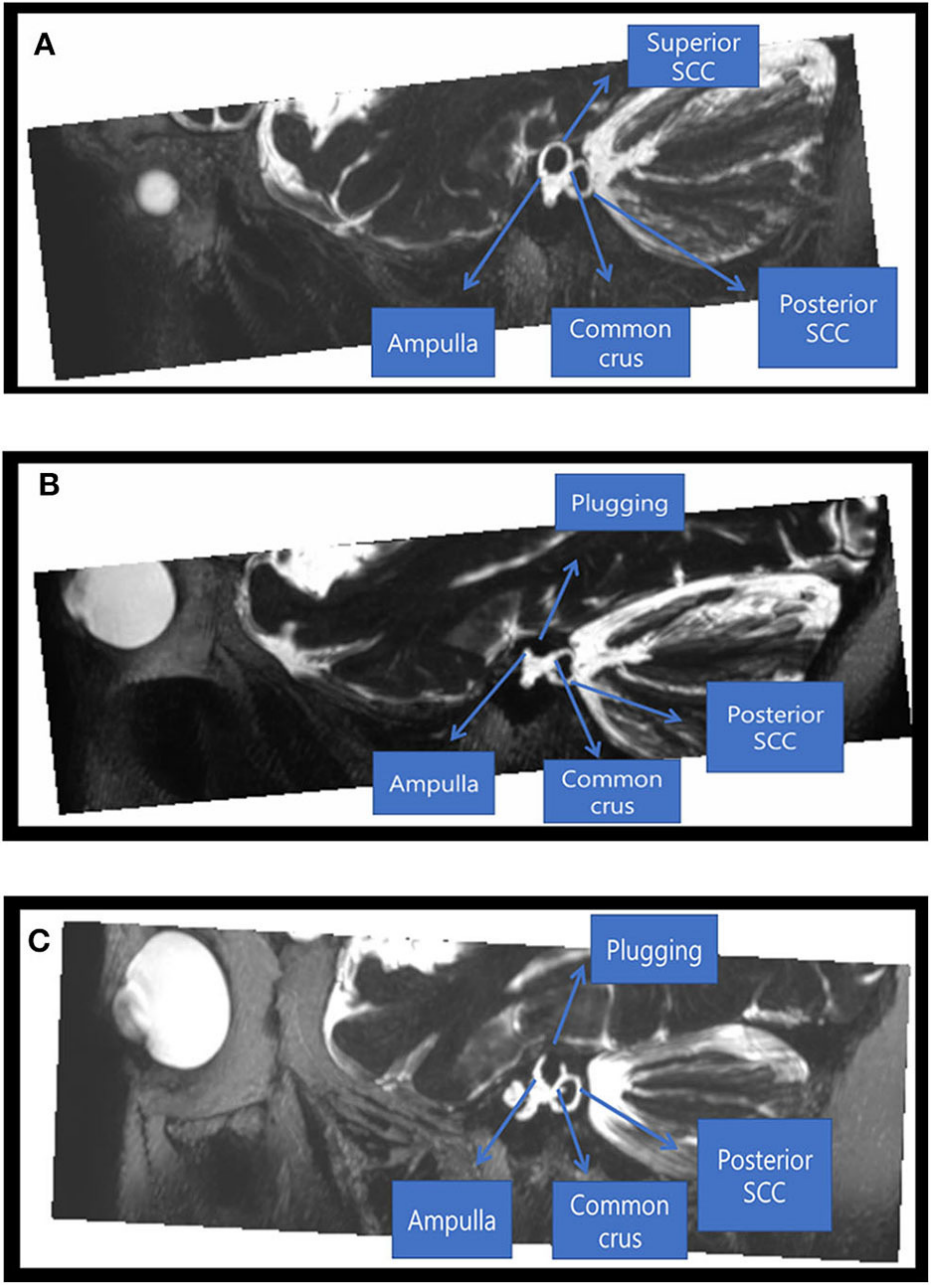
Radiological findings before and after canal plugging. Preoperative **(A)** and postoperative **(B)** images of a 46-year-old male patient who underwent canal plugging. **(A)** Maximum projection intensity reconstruction of oblique sagittal reformatted 3D T2-weighted volume isotropic turbo spin-echo acquisition (T2-VISTA) images (5 mm thick slab) shows T2-bright signal intensity in the patent superior semicircular canal lumen with dehiscence. **(B)** After plugging, the superior semicircular canal lumen was obliterated by filling the defect between the ampulla anteriorly and the common crus posteriorly. **(C)** Postoperative images of a 45-year-old female patient who underwent canal plugging. After plugging, the superior semicircular canal lumen was obliterated by filling the defect between the ampulla anteriorly and the common crus posteriorly.

Postoperative heavily T2-WIs were available for two subjects and showed complete obliteration of T2-bright signal intensity in the patent SC lumen visible on preoperative imaging with filling of the defects at the site of canal plugging. This indicates successful plugging of peri-lymphatic fluid between the anterior and posterior arms (Figure 5). Despite adequate plugging of the SC in these two subjects, one subject (subject 5) had a normal or near-normal VOR gain in the SC and the other (subject 6) showed markedly decreased VOR gains in the plugged SC.

## Discussion

This study explored the postoperative outcomes of individuals with SCD by measuring longitudinal changes in vestibular function after surgical plugging of the dehiscent SC. Consistent with previous reports, individuals with SCD syndrome exhibited marked improvements in terms of subjective symptoms, low-frequency ABGs, cVEMP thresholds, and SP/AP ratios after surgery (2, 16–19). Analyses of repeated quantitative measures of VOR gains over 1 year showed immediate deterioration in VOR gain in the plugged SC that stabilized thereafter in most cases, although VOR gains for the other canals were not affected. Thus, our results suggest that successful plugging of dehiscent SCs is closely associated with a transient, rather than persistent, disturbance of semicircular canal responses exclusively involved in the plugged SC.

It is important to note that we found a propensity for immediate attenuation of VOR gain exclusively in plugged SCs, which suggests that surgical plugging tended to affect the vestibular function of the SC only. Contrary to previous studies (10, 11), VOR gains in the ipsilateral HC and PC were well-preserved in most cases, even immediately after surgery. The plugging procedure led to the elimination of pathologic flow of perilymph in the canal, as evidenced by the complete obliteration of the patent SC lumen by filling the defect at the site of surgical plugging. This finding is consistent with postoperative improvements, which recapitulates the abolishment of the third mobile window effect, including the resolution of associated symptoms, closure of the ABG, and normalization of the cVEMP threshold and SP/AP ratio. In other words, well-preserved VOR gains in the ipsilateral canals, except for plugged SCs, might not be attributed to incomplete compression of the membranous labyrinth (i.e., surgical resurfacing) associated with an increased likelihood of cupula deflection (8). Alternatively, the discrepancy in VOR gains between plugged and non-plugged canals may be associated with a degree in the loss of perilymph during plugging and labyrinthine inflammation.

Unexpectedly, we observed a tendency for a mild decrease in VOR gain in the contralateral PC, although this difference was not statistically significant. A recent study proposed a central compensatory mechanism based on shortened corrective saccade latency and transition from an overt to covert saccade within 1 week after surgical plugging (11). With regard to the presence of refixation saccades, none of the subjects in our cohort had covert and overt saccades during impulses for SC. In this study, nine subjects underwent a video head impulse test immediately after surgery, and five had abnormal VOR gains <0.7 in the plugged SC. Of these subjects, three showed elicitation of compensatory saccades within the first week after surgery; however, the shortening of saccade latency could not be evaluated because of a lack of follow-up during the early phase (Figure S2). Contrary to the higher presence of compensatory saccades on postoperative days 1-2 reported by Mantokoudis et al. (11) the relatively higher interval between the preoperative and first postoperative evaluations in this study (4.2 ± 0.4 days; range, 3–6 days) may hinder the investigation of compensatory saccade metrics. In other words, the shortening of saccade latency and occurrence ration revealed by Mantokoudis et al. (11) could not be evaluated because of a lack of follow-up during the early phase. Overall, these findings suggest that a temporarily reduced VOR in the contralateral PC may have occurred given the loss of anti-compensatory inhibition due to central mechanisms (11). However, further confirmation is needed because several factors, including predictive cues in the brain (20, 21), residual VOR gain (22), vestibular rehabilitation treatment (23), and age (24), may be associated with central compensation. As such, future studies investigating the natural course of VOR gains for plugged canals coupled with compensatory saccade metrics after adjusting for confounders are warranted to determine whether the VOR gains for plugged canals are persistently variable.

**Figure 6:**
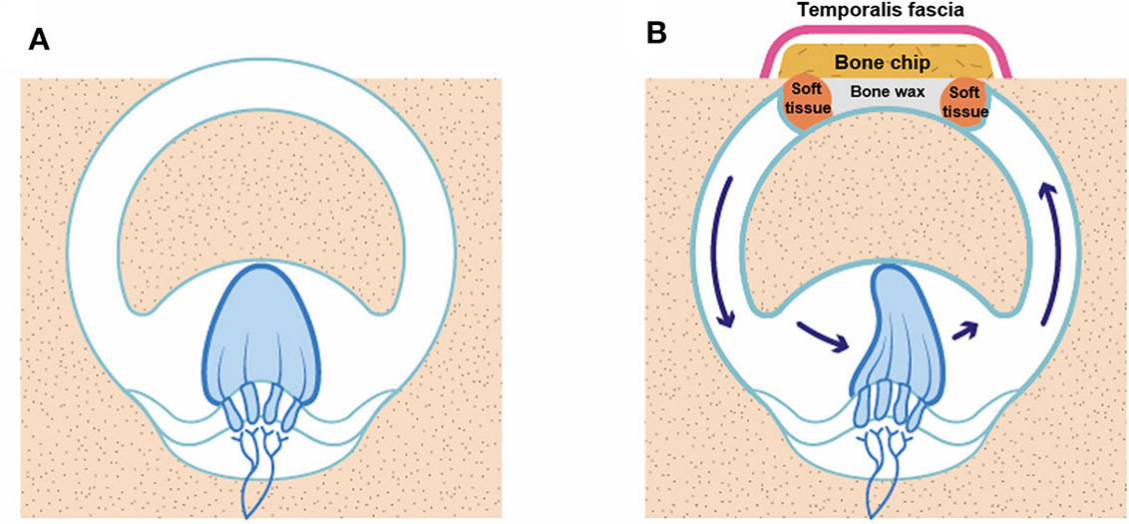
An illustration of how VOR gain is preserved even after canal plugging. **(A)** SCD before surgery, **(B)** SCD after canal plugging. A whole semicircular canal may not be necessary to deflect the cupula on head impulse stimulation.

Our data suggest that the immediate deterioration in VOR gain in the plugged SC was generally a transitory response. The plugged SC gain subsequently stabilized in most cases, which suggests a two-phase natural course of VOR gain for plugged SCs after surgery. As shown in Figure 6, this may be attributable to maintenance of the inertial flow of endolymph across the SC cupula without additional intraluminal fibrosis, despite mechanical obstruction in the middle arm of the SC by surgical plugging. Indeed, previous animal studies have shown that recovery of VOR gain after selective canal plugging could at least be attributable to regained residual sensitivity of the plugged semicircular canals to angular head acceleration (25). Furthermore, it is worth noting that the VOR gain is well-preserved in cases with aplasia or hypoplasia of the horizontal canal or distension of the vestibule, in which cVEMPs and caloric response are not induced (26, 27). Therefore, if not totally absent, trans-cupula inertia may allow cupular displacement by endolymph flow, ultimately normalizing VOR gain via angular rotation. Our findings, coupled with the results of previous studies, support the notion that recovery of SC gain after plugging surgery originates mainly from peripheral recovery processes and changes in the response dynamics of the semicircular canal (i.e., regained trans-cupula inertia) (25, 28). The findings in the animal study further demonstrate that a progressive loss in the VOR gain over the weeks following the additional plugging surgery may be related to fibrosis of the ampulla (25). Given this, adjuvant treatment, such as steroid injections, may be effective in preventing VOR gain from additional intraluminal fibrosis, given the pathologic changes in the inner ear due to the plugging procedure. However, further confirmation is required to clarify these findings. In contrast to our findings, previous studies demonstrated that reduced VOR gain for plugged SCs remained unchanged over time (6, 11). A previous study suggested that global vestibular hypofunction in the immediate postoperative period was largely associated with permanent loss of function over time (10). Similarly, only one subject in the present study showed immediately reduced VOR gain in either the plugged SC or ipsilateral PC after surgery. The reduced VOR gain in the plugged SC then slowly returned to its normal value after ~2 months compared to other subjects who presented with reduced VOR gain in the plugged SC. That is, the discrepancy in recovery patterns of SC gain among studies may be attributable to the degree of functional deterioration in the labyrinth immediately after surgery. In addition, several limitations of the previous studies, such as small sample sizes and incomplete follow-up data, hinder the ability to draw firm conclusions (11).

In the present study, the results of individual analyses demonstrated that the immediately reduced VOR gains in plugged SCs did not necessarily return to normal baseline values even in the long term (Figure 3). Two subjects showed seemingly fluctuating patterns in SC gain over 1 year following surgery. A markedly decreased VOR gain in the plugged SC was found not only immediately after surgery but also at the last follow-up evaluation. However, these subjects did not experience any postoperative complications associated with sensorineural hearing loss. In addition, the normalization of ABGs, SP/AP ratios, and cVEMP thresholds indicated that plugging the dehiscent canal eliminated abnormal pressure transmission via the third mobile window.

In this case series, autophony was the most frequent subjective symptom. Indeed, only one subject did not report autophony before surgery, and this subject had two anterior small tympanic membrane perforations (subject 1). This finding raises the possibility of inserting ventilation tubes to resolve autophony in SCD. Moreover, it is important to take into account clinical manifestations and laboratory findings in patients with SCD complicated by tympanic membrane perforation. Subject 1 had tympanic membrane perforations and a large ABG; however, the SP/AP ration and cVEMP thresholds were within normal range, which can only be expected in SCD. A large ABG may represent the summating effects of tympanic membrane perforation and the presence of a third mobile window. Normal ECoG and cVEMP threshold ranges in this patient can be explained by an increased response of the ECoG and cVEMP threshold by the SCD that was negated by a decreased response due to attenuated mechanical energy delivery through the oval window secondary to tympanic membrane perforation. After plugging, the BC threshold normalized, and the ABG disappeared. However, the SP/AP ratio and cVEMP thresholds remained within the normal range after surgery. The effects of an increased intralabyrinthine response by the SCD and a decreased intralabyrinthine response by the tympanic membrane perforation were similar in this patient.

Moreover, the dehiscence size in those subjects did not differ compared to our subjects. These findings raise the possibility that fluctuating patterns of vestibular function may be due to endolymphatic hydrops in the affected ears (29). Given the higher sample variation in VOR gain in vertical canals, further studies are warranted to validate our findings. Understanding the differential nature of SC gain presenting with fluctuating patterns may provide additional evidence of the necessity for longitudinal and repetitive checkups following surgical plugging in subjects with SCD.

This study has some limitations that should be addressed in future studies. First, it was limited by a relatively small number of subjects and high sample variability of VOR gains, in particular in the vertical canals, which could have potentially led to misinterpretation. Due to the small number of cases involved in video head impulse tests, we identified that our observed SC gains had significant variability and did not exactly follow a Gaussian distribution at each time point (Table S1). Moreover, data on longitudinal changes in VOR gain beyond 12 months are needed to confirm the differential pattern of SC gain after surgery among subjects with SCD. Thus, a study with a longer follow-up period and a larger case series is warranted to validate our observations. Second, the interpretation of the two-phase natural course of plugged SC gain was not specified in detail in the current report. Evidence of central compensation for reduced VOR gain based on the occurrence of corrective saccades and decreased latencies of these corrective saccades over 1 week was recently reported (11). Further studies using the longitudinal changes in VOR gain of our subjects as a reference for parameters affecting central compensations may be important for optimizing individualized vestibular strategies.

Despite these limitations, we have elucidated here the changes in auditory and vestibular function that may be relevant to improving debilitating symptoms after successful canal plugging via the middle cranial fossa approach in subjects diagnosed with SCD. To the best of our knowledge, this study is the first to report the differential natural course of VOR gain between plugged SCs and non-plugged canals after surgical plugging. These findings may have clinical implications with respect to timely and individualized rehabilitation treatment.

## Conclusion

Surgical plugging of dehiscent SCs via the middle cranial fossa approach was an effective treatment option in subjects with SCD. This option ensured that symptoms resolved without significant complications. Indeed, audiometric and electrophysiological findings were normalized after surgery. Specifically, VOR function for plugged SCs decreased immediately after surgical plugging but subsequently normalized in most subjects with SCD. By contrast, non-operated canals tended to remain stable. Understanding the differential natural course of VOR gain, in particular in operated canals, may allow for timely and precise vestibular rehabilitation.

## Data Availability Statement

The original contributions presented in the study are included in the article/Supplementary Material, further enquiries can be directed to the corresponding author.

## Ethics Statement

The studies involving human participants were reviewed and approved by the Seoul National University Bundang Hospital Institutional Review Board (IRB No. B-2004-604-125) and was conducted in accordance with the principles of the Declaration of Helsinki. The patients/participants provided their written informed consent to participate in this study. Written informed consent was obtained from the individual(s) for the publication of any potentially identifiable images or data included in this article.

## Author Contributions

J-WK and S-YL: Conceptualization. S-YL: Methodology, Software, Formal Analysis, Project Administration, Writing? Original Draft Preparation, and Investigation. J-WK: Validation, Resources, Funding Acquisition, and Supervision. S-YL, MK, and YB: Data Curation. J-WK, J-JS, and BC: Writing-Review and Editing. S-YL and YB: Visualization. All authors: contributed to the article and approved the submitted version.

## Conflict of Interest

The authors declare that the research was conducted in the absence of any commercial or financial relationships that could be construed as a potential conflict of interest.

## Supplementary Material

Figure S1 Preoperative and postoperative findings for a 58-year-old female patient with superior canal dehiscence (SCD) and tympanic membrane perforation who underwent superior canal (SC) plugging and type 1 tympanoplasty. **(A)** SCD by the superior petrosal sinus (SPS) shown on high-resolution temporal bone computed tomography images reformatted in the plane of the SC. **(B)** Preoperative tympanic membrane findings. **(C)** Postoperative tympanic membrane findings. **(D)** Pre- and postoperative audiograms.

Figure S2 Video head impulse tests in the plane of the affected superior canal within the first week after surgery (subjects 6, 7, and 8).

Video S1 Surgical plugging for superior semicircular dehiscence via the middle cranial fossa approach.

Table S1 Variability of plugged superior canal (SC) gains at each time point.
