# Supplementary figures and images for: Changes in Vestibulo-Ocular Reflex Gain After Surgical Plugging of Superior Semicircular Canal Dehiscence
Source: Front Neurol. 2020 Jul 21;11:694. doi: 10.3389/fneur.2020.00694 (PMC7385253; doi:10.3389/fneur.2020.00694)

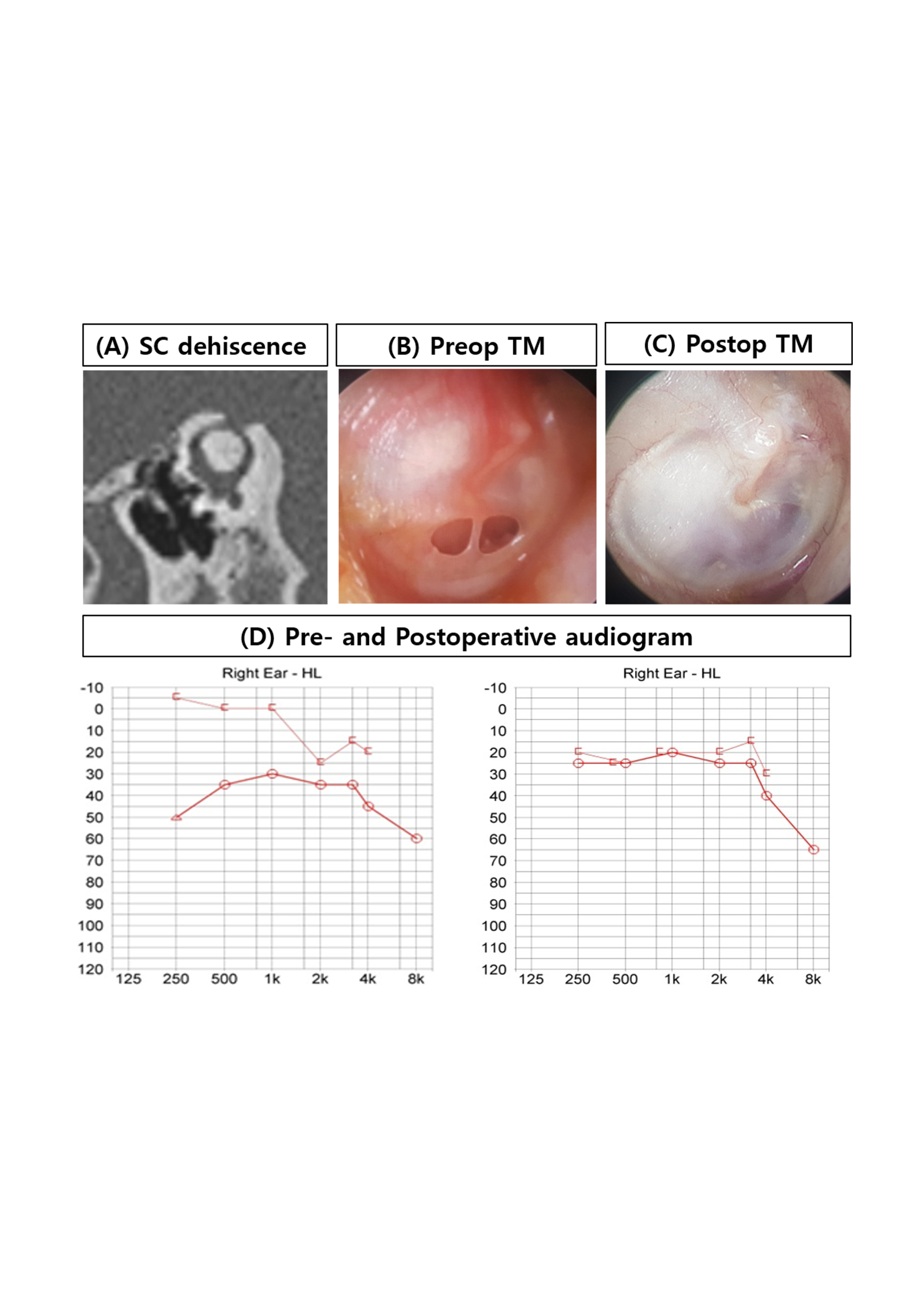

Supplement: Figure S1 — Preoperative and postoperative findings for a 58-year-old female patient with superior canal dehiscence (SCD) and tympanic membrane perforation who underwent superior canal (SC) plugging and type 1 tympanoplasty. (A) SCD by the superior petrosal sinus (SPS) shown on high-resolution temporal bone computed tomography images reformatted in the plane of the SC. (B) Preoperative tympanic membrane findings. (C) Postoperative tympanic membrane findings. (D) Pre- and postoperative audiograms. [file Image_1.TIF]

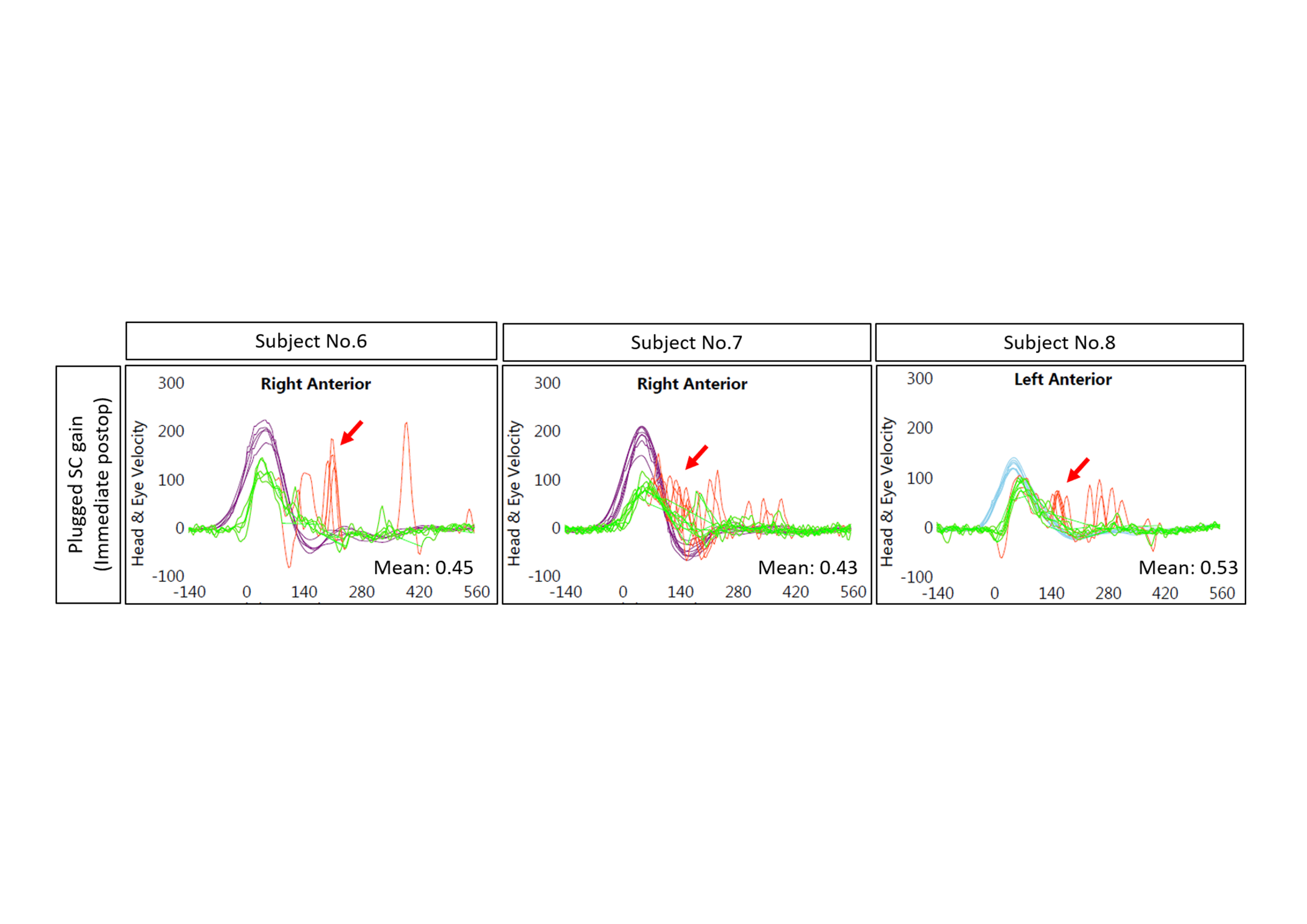

Supplement: Figure S2 — Video head impulse tests in the plane of the affected superior canal within the first week after surgery (subjects 6, 7, and 8). [file Image_2.TIF]
