# Supplementary material for: Changes in Vestibulo-Ocular Reflex Gain After Surgical Plugging of Superior Semicircular Canal Dehiscence
Source: Front Neurol. 2020 Jul 21;11:694. doi: 10.3389/fneur.2020.00694 (PMC7385253; doi:10.3389/fneur.2020.00694)
Supplement: Table S1 — Variability of plugged superior canal (SC) gains at each time point. [file Table_1.DOCX]

Table S1. Variability of plugged superior canal (SC) gains at each time point

|  | Preop  (N=9) | Postop 1st  (N=9) | Postop 2nd  (N=9) | Postop 3rd (N=7) | Postop 4th (N=5) |
| --- | --- | --- | --- | --- | --- |
| Anderson-Darling test |  |  |  |  |  |
| A2* | 1.959 | 0.8396 | 1.214 | 1.742 | 1.260 |
| P value | <0.0001 | 0.0192 | 0.0019 | <0.0001 | 0.0014 |
| Passed normality test (alpha=0.05)? | No | No | No | No | No |
| P value summary | **** | * | ** | **** | ** |
|  |  |  |  |  |  |
| D'Agostino & Pearson test |  |  |  |  |  |
| K2 | 23.85 | 1.696 | 6.450 | 13.10 | 3.332 |
| P value | <0.0001 | 0.4284 | 0.0397 | 0.0014 | 0.1890 |
| Passed normality test (alpha=0.05)? | No | Yes | No | No | Yes |
| P value summary | **** | ns | * | ** | ns |
|  |  |  |  |  |  |
| Shapiro-Wilk test |  |  |  |  |  |
| W | 0.5462 | 0.8096 | 0.7266 | 0.6230 | 0.7309 |
| P value | <0.0001 | 0.0190 | 0.0019 | 0.0001 | 0.0021 |
| Passed normality test (alpha=0.05)? | No | No | No | No | No |
| P value summary | **** | * | ** | *** | ** |
|  |  |  |  |  |  |
| Kolmogorov-Smirnov test |  |  |  |  |  |
| KS distance | 0.3785 | 0.2933 | 0.3443 | 0.3996 | 0.3602 |
| P value | 0.0002 | 0.0148 | 0.0014 | <0.0001 | 0.0006 |
| Passed normality test (alpha=0.05)? | No | No | No | No | No |
| P value summary | *** | * | ** | **** | *** |
